# ADOLESCENT PATIENTS AND THE CLINICAL DECISION ABOUT THEIR HEALTH

**DOI:** 10.1590/1984-0462/;2019;37;4;00011

**Published:** 2019-06-19

**Authors:** Marlene Pereira Garanito, Vera Lucia Zaher-Rutherford

**Affiliations:** a Centro Universitário São Camilo, São Paulo, SP, Brazil.

**Keywords:** Adolescence, Decision making, Personal autonomy, Bioethics, Adolescência, Tomada de decisões, Autonomia pessoal, Bioética

## Abstract

**Objective:**

To carry out a review of the literature on adolescents’ participation in decision making for their own health.

**Data sources:**

Review in the Scientific Electronic Library Online (SciELO), Latin American and Caribbean Health Sciences Literature (LILACS) and PubMed databases. We consider scientific articles and books between 1966 and 2017. Keywords: adolescence, autonomy, bioethics and adolescence, autonomy, ethics, in variants in the English, Portuguese and Spanish languages. Inclusion criteria: scientific articles, books and theses on clinical decision making by the adolescent patient. Exclusion criteria: case reports and articles that did not address the issue. Among 1,590 abstracts, 78 were read in full and 32 were used in this manuscript.

**Data synthesis:**

The age at which the individual is able to make decisions is a matter of debate in the literature. The development of a cognitive and psychosocial system is a time-consuming process and the integration of psychological, neuropsychological and neurobiological research in adolescence is fundamental. The ability to mature reflection is not determined by chronological age; in theory, a mature child is able to consent or refuse treatment. Decision-making requires careful and reflective analysis of the main associated factors, and the approach of this problem must occur through the recognition of the maturity and autonomy that exists in the adolescents. To do so, it is necessary to “deliberate” with them.

**Conclusions:**

International guidelines recommend that adolescents participate in discussions about their illness, treatment and decision-making. However, there is no universally accepted consensus on how to assess the decision-making ability of these patients. Despite this, when possible, the adolescent should be included in a serious, honest, respectful and sincere process of deliberation.

## RESUMO

**Objetivo:**

Realizar uma revisão da literatura sobre a participação do adolescente na tomada de decisão sobre a sua saúde.

**Fonte de dados:**

Revisão nos bancos Scientific Electronic Library Online (SciELO), Literatura Latino-Americana e do Caribe em Ciências da Saúde (LILACS) e PubMed. Consideramos artigos científicos e livros entre 1966 e 2017. Palavras-chave: *adolescência, autonomia, bioética e adolescência, autonomia, ética*, em línguas inglesa, portuguesa e espanhola. Critérios de inclusão: artigos científicos, livros e dissertações que contemplassem a tomada de decisão clínica pelo paciente adolescente. Critérios de exclusão: relatos de caso e artigos que não abordavam a questão norteada nesta pesquisa. Do total de 1.590 resumos, 78 foram lidos na íntegra, e 36, utilizados neste manuscrito.

**Síntese dos dados:**

A idade em que o indivíduo é capaz para tomar decisões é motivo de debate na literatura. O desenvolvimento de um sistema cognitivo e psicossocial é um processo demorado, e faz-se fundamental a integração da investigação psicológica, neuropsicológica e neurobiológica na adolescência. A capacidade de reflexão madura não é determinada pela idade cronológica; em teoria, um menor maduro seria capaz de consentir ou recusar um tratamento. A tomada de decisão exige análise cuidadosa e reflexiva dos principais fatores associados, e a abordagem desse problema deve ocorrer por meio do reconhecimento da maturidade e da autonomia que existe no adolescente. Para tanto, é necessário “deliberar” com ele.

**Conclusões:**

Diretrizes internacionais recomendam que os adolescentes participem de discussões sobre sua doença, tratamento e tomada de decisão, entretanto não há nenhum consenso universalmente aceito sobre como avaliar a capacidade de decisão desses pacientes. Apesar disso, quando possível, o adolescente deve ser incluído em um processo sério, honesto, respeitoso e sincero de deliberação.

## INTRODUCTION

Medical care for teenage patients is complex due to the peculiar characteristics of the teenage stage of life and the new type of doctor-patient relationship that begins.

With the start of the teenage years, the relationship between physicians and a parent/guardian, which up until then has occupied a prominent place in the dynamics of medical consultations, begins to change with the effects of this natural phenomenon. The relationship between physicians and their clients should give space for this new phase, establishing a more complex dynamic: doctor-adolescent-parents/guardians.

From a medical point of view, this new stage results in new challenges, especially those related to ethical issues. Among them, we highlight the possibility of emerging conflicts during the clinical care of adolescents with serious diseases, especially regarding the participation of the adolescent in the decision-making process regarding their treatment.

The legal ambiguity associated with the clinical difficulty in evaluating adolescent patients adds to the fact that there are no publications, especially Brazilian ones, regarding adolescent patients’ role in the decision-making process. This creates challenges for bioethics with regard to giving due ethical respect to the autonomy of the subjects in these specific conditions. Thus, this research aims to perform a literature review on adolescent participation in the decision-making process regarding his or her own health.

### Data sources

The literature review was performed in the following electronic databases: Scientific Electronic Library Online (SciELO), Latin American and Caribbean Literature in Health Sciences (LILACS) and PubMed - National Library of Medicine of the National Institutes of Health.

Scientific articles, books and dissertations published between 1966 and December 2017 were considered. The keywords used were: *adolescence*, *autonomy*, *bioethics* AND *adolescence*, *autonomy*, *ethics*, included in the Descriptors in Health Sciences (DeCS) in English, Portuguese and Spanish.

The inclusion criteria used in the selection were: scientific articles, books and dissertations that contemplated the theme of adolescent patients’ autonomy in the clinical decision-making process. Cases and articles that did not deal with the issue addressed in this study were excluded.

**Figure 1:**
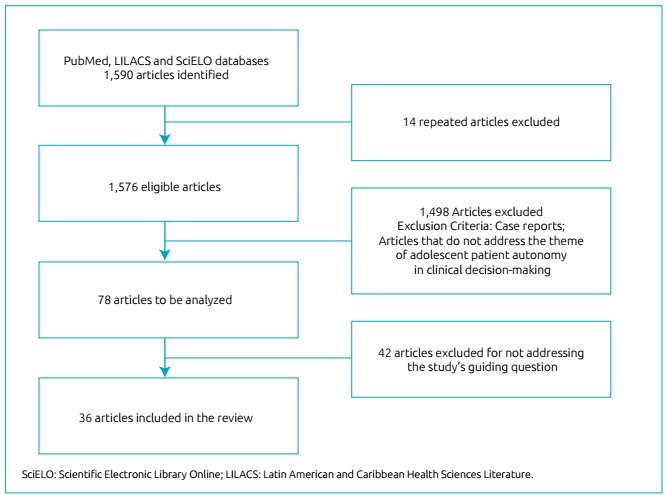
Flowchart of the research.

Of the total of 1,590 publications, 14 were duplicates, that is, were present in more than one database. After reading all of the abstracts, 82 publications were selected for the reading and analysis of their contents in full, and 36 were used in this manuscript (Figure 1). The selected publications were critically evaluated by two authors. Doubts about the selection of articles were studied and discussed among the researchers until agreement was reached.

### The decision-making process

Decision making can be defined as the process of choosing between two or more competing alternatives, requiring a cost and benefit analysis of each option and estimating their consequences in the short, medium and long term. Since the results of our decisions are uncertain, it can be said that decision-making involves risk analysis. This process is closely related to the ability to control impulses and impulsivity, and involves the ability to assess consequences in the medium and long term. Therefore, it can be considered that decision-making is indispensable for the social adaptation of the individual, and particularly difficult when there is a greater need to weigh rewards and/or immediate and future losses.^1^

According to Lorda, the following requirements are fundamental in order to make decisions autonomously:

- Lack of external coercion that significantly restricts one’s freedom to decide.
- True information about the elements involved in the decision-making process.
- Ethical and legal recognition, in sufficient level of psychological abilities, that allows one to perform the mental process of deliberation.
- An appropriate degree of life experience that nurtures wisdom and prudence in the deliberation process.
- Environment (family, social, economic, political, cultural, etc.) that allows one to develop one’s possibilities as a deliberative subject in an active and positive way.^2^

### Neurobiological and neuropsychological development during adolescence

Recent discoveries in the field of developmental neuroscience have stimulated scientific interest in the study of brain development during adolescence, as well as considerable speculation about the connections between brain maturation and the intellectual, behavioral, and emotional development of adolescents.^3^

Throughout the course of adolescence, significant changes occur in various regions of the brain. One theory is that teens have a model for two decision-making systems. It is believed that a socio-emotional system, located in the limbic and paralimbic regions of the brain, develops around puberty, with increased dopaminergic activity, and manifests itself as reward-seeking behavior. The cognitive control system, which promotes self-regulation and impulse control, is located in the prefrontal cortex. The changes that occur in the processes of myelination and synapse in the prefrontal cortex corroborate the improvement of information efficacy and its processing, long-term planning, self-assessment, self-control, coordination of affection, cognition, and risk and reward accuracy.^3^

According to Almeida,^4^ these changes occur at different times for the different regions of the brain, from the posterior to the anterior, continuing until the beginning of the third decade of life. The last regions in which the reorganization process is completed are the dorsolateral frontal cortex, which is responsible for impulse inhibition, action planning and abstract thinking; and the orbitofrontal cortex, which takes care of moral judgments and the emotional information that underlies the decision-making processes.^5^ ^,^ ^6^

The relationship between cognitive development and the development of judgment, decision-making, and risk taking is a new direction of adolescent research. As such, there is a hypothesis that adolescents 16 years of age or older share the same logical competences of an adult, but differences in social and emotional factors that lead to differences in real decision-making.^3^

In this regard, a psychosocial survey suggests significant differences between maturity levels between adolescents and adults. The decision-making capacity of adolescents as a group is between that of young children and that of adults, and, considering this, involvement in health decision-making processes should also occur at a similar level.^7^

In addition, physical alterations and emotional and mental disorders must be taken into account, since they can compromise the appreciation and rationality of the decisions to be made. It is worth mentioning that disadvantaged segments of the population, which are more vulnerable due to their scarce resources and the lack of opportunities available to them, face various restrictions on full participation in socioeconomic and cultural life, yet they should fully enjoy the rights of any human being and citizen, and thus be treated and considered, respecting their autonomy in decision-making.

Steinberg also draws attention to the fact that the increase in the search for sensations, risk-taking and reckless behavior in adolescence are influenced by puberty, not by chronological age.^3^ Puberty is characterized by the period of biological changes that consists of the maturing of the physical body and the beginning of reproductive ability.^8^ The ages at which pubertal events occur have wide individual variation, occurring generally between the ages of 8-12 years old for girls and 9-13 years old for boys.^9^

Due to great discoveries in the areas of neuroscience and neuroimaging, it was possible to study a series of mechanisms in the circuitry and maturation of the brain that help to formulate explanations and hypotheses in order to understand some adolescent behaviors. All of this becomes essential to understand adolescents’ inclination toward risk behaviors, but it is not enough to explain its occurrence nor the behavioral variations between individuals on its own, since any decision-making involves not only a neurobiological process, but also interactions with ambient aspects: familiar, social, economic, emotional and cultural.^10^

With regard to cognition and emotion, there is an increasing understanding that this interaction also unfolds in other directions and in important aspects, like the fact that emotion has a significant impact on basic cognitive processes, including decision-making. As such, as these feelings develop and as this interaction occurs, there is reason to study them in the field of affective neuroscience.^3^

Children, between 6 to 11 years of age, are able to perform concrete thinking, which extends to the understanding of others and to the consequences of many of their actions.^11^ ^,^ ^12^

In general, up to the age of 11 and 12, children see their parents, teachers and physicians as authority figures, ie. people who represent power, safety, and promote social well-being. Because of this view, they tend to conform to the demands of these figures, but as they grow older, they become less susceptible to such influences. Adolescents become progressively more capable of asking questions and resisting external pressures, and from the age of 14, most are able to make rational choices in many different contexts.^12^

In adolescence, new cognitive skills are acquired and referred to by Piaget as hypothetical-deductive reasoning, that is, the ability to think of hypothetical solutions and formulate a systematic plan to deduce which of the solutions is correct. Exercising this new ability leads to the greater development and differentiation of operational thinking, and gradually this form of thinking fits into reality.^13^ However not all adolescents or adults reach this level of operational thinking, since both the maturation of the nervous system as well as sufficient experiences or opportunities to interact with one’s environment are needed.^14^

At the age of 15, a strong development of metacognitive understanding emerges, including knowledge of one’s own qualities, characteristics, and limitations with regard to decision-making. In this way, Coa and Pettengill consider that, from the age of 15 onwards, adolescents achieve basic competences to exercise their autonomy fully, and it is up to the people involved in the socialization process of the child to help them in the development of their autonomy.^11^

In addition to cognitive abilities, the ability to think/act is related to life experience. Therefore, adolescents living with serious illnesses may exhibit a better understanding of the aspects involved in their treatment, compared to individuals of the same age who do not have experience with the chronic disease.^13^

Considering the above, the age at which an individual becomes able to make decisions has generated wide debate in the literature. The question is that the development of an integrated, cognitive and psychosocial system is a time-consuming process, and to understand it in the context of adolescence, the integration of psychological, neuropsychological and neurobiological research is fundamental.^3^

Given this integrated system, it is up to the medical team to interact with adolescents, respecting the different stages of their development. A physician-patient relationship that maintains this coherence and has good communication between the two parties is essential for adolescents to feel encouraged to participate in their own care.^15^

### Decision-making

The ability to make decisions is difficult to evaluate and is linked to understanding and maturity.^16^ ^,^ ^17^ ^,^ ^18^ It should be seen as a similar process to that of other aspects of development.^19^ A proposal to assess decision-making capacity assumes that the patient meets the following conditions in order to make a decision: the ability to understand the information relevant to the decision (the ability to understand the medical problem, the ability to understand the proposed treatment, the ability to understand the alternatives - when there are alternatives - for the proposed treatment, the ability to understand the option to refuse or withdraw treatment, the ability to evaluate the reasonably foreseeable consequences if they accept or refuse the proposed treatment, the abilty to make a decision that is substantially based on delusions or depression), the ability to deliberate on their choices according to their values and personal goals, and the ability to communicate (verbally or non-verbally) with caregivers.^16^ ^,^ ^20^

In this way, mature reflection capacity is not determined by chronological age; in theory, a mature minor that is able to consent to treatment, also has the right to refuse it.^16^ ^,^ ^21^ Mature minor is a term used to designate minors from a legal standpoint, but who have sufficient capacity to engage in medical and other decision-making processes.^22^ ^,^ ^23^ Thus, decision-making cannot consist of a mathematical equation without the careful and reflexive analysis of the main factors associated with it. ^24^

Under these circumstances, health care providers are usually responsible for assessing the young patient’s ability and to what extent and situation the patient can make a decision about his or her health.^25^ ^,^ ^26^. However, often, the involved parties do not have clarity about their decision-making capacities.

Health professionals have always done such ability assessments based on a mix of clinical experience, prudence, and common sense, and they have been welcomed and accepted by family members.^27^ In this respect, it is necessary to develop a methodology for assessing ethical, legal and scientifically sound capabilities. However, Simón^27^ points out that, even if a method for capacity analysis was established, it would not be infallible. It would not have a sensitivity or specificity of 100%, since the clinical judgment that a patient needs, as scientific as it may seem, involves deliberative and prudential moral judgment. Nonetheless, this would not be incompatible with the effort to rationalize ability assessment procedures, with the aim of increasing accuracy. In this context, a good questioning, directed according to the selected capacity criteria, and a quality clinical interview are tools that can provide a lot of information.

### Legal aspects

From a legal point of view, adolescents under the age of 16 years old are completely incapable of performing acts of civil life (Article 3 of the Brazilian Civil Code), and those over 16 and under 18 are relatively incapable of certain acts (Article 4 of the Brazilian Civil Code), which means they can perform them with the assistance of their legal guardians or with judicial authorization, in case that they differ from or lack parents or legal guardians. Adolescents are allowed to vote at age 16 and choose their own sexual and reproductive life. On the other hand, it is interesting to require an adolescent’s (age 12 or older) consent to be placed in a substitute family, while that same person cannot perform other acts of civil life, without the authorization of both parents or their legal representatives.^28^ ^.^ ^29^

In order to empower adolescents in the deliberation process, proposals have been developed regarding their decision-making capacity. In this regard, an alternative form of adulthood, related to the decision-making process about one’s own body and health was proposed by Mônica Aguiar in 2012, and it is called bioethical majority. For the author, based on article 28, paragraphs 1 and 2, of the Statute of the Child and Adolescent (*Estatuto da Criança e do Adolescente* - ECA), the age of 12 represents an adequate age to presume an adolescent has complete ability to act with regard to their right to life and health. This could be a feasible solution, because it is close to the current status quo and gives an adolescent the full capacity to make choices about his or her own body and health. According to this proposal, the questions concerning the life and health of a person must be decided by that person, even when they have not reached a legal age.^29^

### Bioethics and decision-making

Although numerous theories and norms on capacity have been proposed in the literature, bioethicists Allen Buchanan and Dan Brock proposed in 1990 that the capacity for decision-making varies according to the complexity of the medical options available in conjunction with the patient’s ability to deal with such a situation. In other words, high risk situations require a certain standard of ability, while a lower standard can be accepted when the risk is relatively minor. Furthermore, according to this theory, the level of decision-making ability is not static, and may change even in a single moment of medical care and, therefore, it should be reevaluated during the course of the disease. Considering this, Hein points out that the level of risk (high or low) and the complexity of decisions (high or low) are not quantifiable and should therefore be better studied.^13^

In practice, arguments regarding a patient’s ability rarely arise unless there is a disagreement about values. When a patient agrees with the doctor’s recommendations, his or her ability to understand, decide, and consent to treatment is rarely examined, as the true thought process of the adolescent is not questioned. In general, the situations when a patient’s capacity is questioned are those in which there is a conflict between the patient’s will and the doctor’s judgment as to the patient’s best interest. In this regard, Gracia points out the great mistake of paternalism: to consider all people who have a value system different from ours to be immature or incapable.^24^

According to Grace, the subject’s approach in terms of maturity is wrong and will never lead to a correct solution. The problem is not whether the young person is mature or not. In principle, in many cases, the young person is as mature as they will be later in life or as mature as many other adult citizens, to whom no one questions their autonomy to make decisions. However, in other cases, the adolescent is not always as mature as they will be later in life, when his or her process of personal maturation has reached new levels.^32^

The approach of this problem must occur through the recognition of the maturity and the autonomy that adolescents have. Therefore, it is necessary to “deliberate” with the adolescent. The key is to include them in a serious, honest, respectful and sincere process of deliberation. The deliberation process with a young person is especially complex, because it requires great maturity in who leads the process.^30^ In this regard, in the case of chronic diseases of great severity and / or other issues of strong social impact, the time given to process the information is very helpful in the maturation process of everyone involved.

The issue of adolescent maturity reveals the problem of adult maturity (family members, educators, professionals, etc.). It is a well-known principle in human relations theory that no one can help other people in conflicts that they themselves have not resolved. Helping relationships require great psychological maturity, and when they are putting forth moral problems, they also need proper moral development.^32^

Maybe one of the serious problems that our health system has right now is the lack of professional training in ethical issues, which means that, in general, health professionals do not know how to deal with this type of conflict. Furthermore, pressured by their own anguish, they assume extreme attitudes, ranging from rejection of the patient to the trivialization of the problem. ^32^ In truth, this approach has as undercurrent of genuine “clinical paternalism”, that is, the best thing for the patient is established according to a medical point of view. Taking this route means continuing with an authoritarian relationship, because you take away the opportunity for sick people to make decisions about themselves, about their living conditions and about the way they want to live with the disease.^33^ ^,^ ^34^

## FINAL CONSIDERATIONS

Although international guidelines recommend that adolescents participate in discussions about their illness, treatment, and decision-making, there is no universally accepted consensus on how to assess an adolescent patient’s decision-making abilities. ^35^ ^,^ ^36^ Nevertheless, when possible, adolescent patients should be included in a serious, honest, respectful and sincere process of deliberation.
